# Bio-Separated and Gate-Free 2D MoS_2_ Biosensor Array for Ultrasensitive Detection of BRCA1

**DOI:** 10.3390/nano11020545

**Published:** 2021-02-21

**Authors:** Yi Zhang, Wei Jiang, Dezhi Feng, Chenguang Wang, Yi Xu, Yufeng Shan, Jianlu Wang, Ziwei Yin, Huiyong Deng, Xianqiang Mi, Ning Dai

**Affiliations:** 1 State Key Laboratory of Infrared Physics, Shanghai Institute of Technical Physics, Chinese Academy of Sciences, Shanghai 200083, China; zy_scube@163.com (Y.Z.); 13681827797@163.com (W.J.); jlwang@mail.sitp.ac.cn (J.W.); yinziwei@mail.sitp.ac.cn (Z.Y.); 2 School of Electronic Electrical and Communication Engineering, University of Chinese Academy of Sciences, Beijing 100049, China; 3 Shanghai Advanced Research Institute, Chinese Academy of Sciences, Shanghai 201210, China; fengdzh2020@163.com (D.F.); wangcg@sari.ac.cn (C.W.); xuyi@sari.ac.cn (Y.X.); 4 Hangzhou Institute for Advanced Study, University of Chinese Academy of Sciences, Hangzhou 310024, China; shanyufeng@mail.sitp.ac.cn; 5 CAS Center for Excellence in Superconducting Electronics (CENSE), Shanghai 200050, China; 6 Key Laboratory of Functional Materials for Informatics, Shanghai Institute of Microsystem and Information Technology, Chinese Academy of Sciences, Shanghai 200050, China

**Keywords:** chemical vapor deposition, MoS_2_ sensor arrays, DNA tetrahedron probe, bio-separated sensing part, gate-free structure, reusability

## Abstract

2D molybdenum disulfide (MoS_2_)-based thin film transistors are widely used in biosensing, and many efforts have been made to improve the detection limit and linear range. However, in addition to the complexity of device technology and biological modification, the compatibility of the physical device with biological solutions and device reusability have rarely been considered. Herein, we designed and synthesized an array of MoS_2_ by employing a simple-patterned chemical vapor deposition growth method and meanwhile exploited a one-step biomodification in a sensing pad based on DNA tetrahedron probes to form a bio-separated sensing part. This solves the signal interference, solution erosion, and instability of semiconductor-based biosensors after contacting biological solutions, and also allows physical devices to be reused. Furthermore, the gate-free detection structure that we first proposed for DNA (BRCA1) detection demonstrates ultrasensitive detection over a broad range of 1 fM to 1 μM with a good linear response of R^2^ = 0.98. Our findings provide a practical solution for high-performance, low-cost, biocompatible, reusable, and bio-separated biosensor platforms.

## 1. Introduction

Recently, more and more diseases show characteristics of being difficult to detect in early stages and difficult to cure in late stages [1,2]. Therefore, early, rapid, and accurate diagnosis has attracted widespread public attention. In order to meet the needs of real-time ultra-low concentration and accurate detection, scholars in the life sciences, physics, chemistry, and other major fields have devoted themselves to research on electrochemical reactions [3,4], surface potential [5,6], and bioelectronic signal of biosensors [7,8]. Thin film transistors (TFTs), due to their signal sensitivity, high openness, miniaturization, and compatibility with other systems, have developed many applications in biological detection of proteins [9,10], DNA [11,12] and RNA [13,14]. Molybdenum disulfide (MoS_2_), a mature two-dimensional material, has been proven successful in these applications due to its intrinsic energy gap, nano–bio hybrid ability, synthesis controllability, low cost and complementary metal oxide semiconductor (CMOS) compatibility [15,16,17]. However, in view of these characteristics, researchers have achieved detection of various biotargets at femtomolar concentrations but rarely focus on the biocompatibility (solution erosion) and reusability of the MoS_2_-based TFT biosensors [18,19]. The surface modification strategy in early reports limits the biomodification area in the sensing area since the signal reaction principle of TFT biosensors requires that the gate part must be chosen as the sensing area [20]. Unfortunately, as he TFT biosensors are extremely sensitive to humidity and oxygen and because the bio-solution is extremely corrosive to the TFTs, the biosensors are prone to generating false signals and are easily corroded by bio-solutions. Many efforts, including increasing the thickness of the gate dielectric and reducing the concentration of the salt solution, have been made to improve these problems, but these schemes have not fundamentally solved the problem of biocompatibility and reusability [21,22]. Therefore, separating the sensing area from the TFTs to realize the dry and wet separation is an urgent problem. Recently, the device structure of separative extended gate has been proposed in III/IV compound semiconductor-based biosensors that extended their service life, but the manufacturing cost was high and the manufacturing process was complicated [23]. Additionally, the biomodification method that has been used was not very efficient and, more specifically, the device exhibited poor biotarget performance [24]. Therefore, in order to achieve better biocompatibility in MoS_2_-based TFT biosensors and ensure a rapid, robust, high performance of target hybridization, it is necessary to develop a simple and effective biomodification method and simplify the process of fabrication, lower the cost, and improve the reliability of a large-scale array of MoS_2_ TFTs.

In this work, we fabricated a bio-separated and gate-free structured MoS_2_ TFT biosensor to detect DNA (BRCA1). The device consists of two separated parts, i.e., MoS_2_ TFT arrays and a sensing part. A simple-patterned chemical vapor deposition (CVD) growth method was developed to fabricate the array, and a one-step biomodification of the sensing pad based on the DNA tetrahedron probes (DNA-TPs) was developed to simplify the modification process and improve the stability of the probe. Table 1 presents the performance compared to other state-of-the-art detection methods for DNA detection. The real-time detection of BRCA1 response demonstrated that our bio-separated and gate-free sensor arrays indicated ultrasensitive detection over a broad range of 1 fM to 1 μM, with a good linear response of R^2^ = 0.98, which is better than the previously reported results. The combination of a rapid reliable biomodification sensing pad and simply fabricated MoS_2_ TFT arrays with gate-free structures meet the biocompatibility, reliability, and ultrasensitive detection of the biotarget, which exhibited a practicable development of high-performance, low-cost, biocompatible, reusable, and bio-separated biosensor platforms.

## 2. Materials and Methods

### 2.1. Biomaterials

All of the DNA sequences were synthesized by Sangon Biological Engineering Technology & Services Co. Inc. (Shanghai, China). The DNA sequences of tetrahedron probe are shown in Table S1 in the Supplementary Materials, which were synthesized based on our previous works [32,33,34]. The detected DNA in use was as follows:

Target detection (BRCA1): 5′-GAACAAAAGGAAGAAAATC-3′,
Mismatch DNA sequence: 5′-TGCAAGGTGTCAGTATAATCCGACGTTTT-3′.

3,3′,5,5′-tetramethylbenzidine (TMB, H_2_O_2_ included) was provided by Sigma-Aldrich (St. Louis, Mo, USA). Tris (2-carboxyethyl) phosphine hydrochloride (TCEP) and other chemical reagents were purchased from Sinopharm Chemical Reagent Co. Ltd. (Shanghai, China).

### 2.2. Synthesis of Patterned MoS_2_ and Fabrication of MoS_2_ Device Arrays

**Figure 1:**
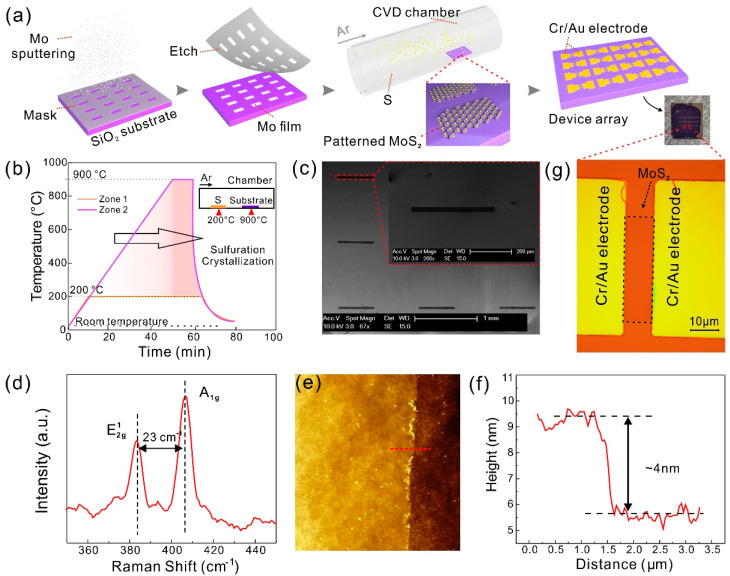
The synthesis and characterization of few-layer MoS_2_ films and device arrays. (**a**) Schematic diagram of patterned MoS_2_ chemical vapor deposition (CVD) growth process. (**b**) The growth temperature curve of different zones, and the inset is a schematic diagram of the position of the sulfur powder (Zone 1) and the substrate (Zone 2). (**c**) SEM images of the grown patterned MoS_2_ film array, the scale bars are 1 mm and 200 μm (inset), respectively. (**d**) Typical 514 nm laser Raman spectra of MoS_2_ films. (**e**) Surface topography of MoS_2_ films examined by atomic force microscopy (AFM), and the height profile along the red line shown in (**f**). The thickness of the MoS_2_ film is 4 nm. (**g**) The optical image of the unit device. The area of the MoS_2_ channel is 10 × 40 μm^2^ and the scale bar is 10 μm.

The patterned MoS_2_ film arrays were synthesized in a two-temperature–zone CVD chamber. The detailed preparation process is as follows. Firstly, a pattern mask was formed on a SiO_2_/Si substrate using UV lithography. Secondly, Mo was sputtered by ion beam sputtering via the mask to form Mo film arrays. Thirdly, the SiO_2_/Si substrate with Mo film arrays and S powder (Alfa Aesar, 99.999%, adequate), as sources, were placed successfully on the quartz boat in each temperature zone. After evacuating the chamber and passing a large flow of Ar (99.999%) gas for 20 min, 100 sccm Ar (99.999%) gas was used as a carrier gas. During the growth, the pressure of the CVD chamber was maintained at 1 Torr. The S powder (Zone 1) and substrate (Zone 2) were simultaneously heated in accordance with the process in Figure 1b. The temperature of Zone 1 was heated to 200 °C within 10 minutes and maintained for 50 minutes to ensure S powder evaporated into S vapor. At the same time, Zone 2 was risen to 900 °C in 50 min and kept for 10 min. The patterned MoS_2_ film arrays were formed through gradual sulfuration, recrystallization, and stabilization at high temperatures. Finally, the chamber was quickly cooled to room temperature by flowing Ar gas.

After forming the patterned MoS_2_ film arrays, the electrodes were directly patterned by laser direct writing lithography, then 15 nm/45 nm Cr/Au were deposited on MoS_2_ by thermal evaporation. Followed by the lift-off process, the MoS_2_ device arrays, with a channel length and width of *L* = 10 μm and *W* = 40 μm, were obtained for BRCA1 detection.

### 2.3. Synthesis of DNA-TPs

The DNA-TPs were synthesized as follows: 2 μL tetra-sequence (50 μM) was mixed with 10 μL TCEP (30 μM) and 82 μL TM buffer (20 mM Tris, 50 mM MgCl_2_, pH = 8.0), respectively. The mixed solution was then heated to 95 °C holding for 10 min. The final DNA-TP solution (100 μL, 1 μM) was obtained after being cooled to 4 °C over 30 s, using a PTC-100™ thermal cycler (Bio-Rad Laboratories, Inc., Hercules, CA, USA). The DNA-TP solution was stored at 4 °C for further experimental processes.

### 2.4. Development of Separable Biosensor

Prior to modification, 15 nm Cr and 45 nm Au were deposited in a sequence on a SiO_2_/Si substrate by thermal evaporation, yielding four round gold electrodes (d = 3 mm) with an interval space of 2 mm via metal shadow mask. The bio-separated sensing part was obtained. Afterwards, 20 μL DNA-TPs was evenly distributed among the 4 gold electrodes and incubated for 12 hours in a humidity chamber at room temperature to form Au-S bonds on the surface of gold electrodes. After that, these gold electrodes biomodified by the DNA-TPs were rinsed in ultrapure water (ρ = 18.2 MΩ·m) and dried with N_2_ gas for *I-t* further target detection. Next, 5 μL of 1 fM, 1 pM, 1 nM, and 1 μM BRCA1 were dropped on the 4 functionalized gold electrodes in sequence and hybridized for 1 h in a humidity chamber at room temperature, which is based on the study of DNA hybridization kinetics and previous experiments [12,30]. After completing the hybridization, the bio-separated sensing part was rinsed with ultrapure water and dried with N_2_ gas. Finally, the sensing part was connected to the MoS_2_ device arrays part via Au wire to form a separable biosensor array, at which point the response of the current signal would be detected by the MoS_2_ device. 

### 2.5. Measurement Instruments

Surface topography was observed by Dimension FastScan Bio (atomic force microscopy (AFM)) (Bruker Corp., Billerica, Germany) using tapping mode, and Nova Nano SEM 450 (FEI Company, Hillsboro, OR, USA). Raman spectroscopy measurements were performed using Lab Ram HR800 (Horiba, Ltd., Kyoto, Japan) with a 514 nm laser. The binding energy was measured by ESCALAB 250XI (XPS) (Thermo Fisher Scientific Inc., Waltham, MA, USA). The measurements of electrical characterizations were performed by a Keithley 2602 source meter (Keithley Instruments LLC., Solon, OH, USA).

## 3. Results and Discussion

### 3.1. The Fabrication of MoS_2_ TFT Arrays

Most of the reported MoS_2_-based biosensors are unit devices, which is just exploratory work for the laboratory, due to the small size, irregular shape, and low density of the MoS_2_ film obtained through mechanical microexfoliation [35]. Efficient and reliable large-scale growth of MoS_2_ films is essential to improve the consistency of materials and substrate utilization. The typical CVD growth and sequential solvent exchange method have been utilized to obtain large-area MoS_2_ and reduce the fabrication of MoS_2_-based biosensors [36]. However, there are still no reported MoS_2_ array devices based on CVD growth for biotarget detection. We demonstrated a patterned MoS_2_ film array synthesized by the three-step CVD method. The detail is presented in the Experiment Section. The patterned MoS_2_ film array forms after sulfuration, recrystallization, stability, and rapid cooling during the heating process. The SEM image in Figure 1c and its inset exhibit that the growth of the MoS_2_ film array on the substrate is consistent with the desired results. In addition, Raman spectroscopy and atomic force microscopy (AFM) are useful and basic techniques to characterize the quality of growth material. Raman spectra displayed in Figure 1d show the lattice vibration modes of MoS_2_ film. Two distinguishing peak frequencies around 383.6 cm^−1^ and 384.4 cm^−1^ correspond to $E_{2g}^{1}$ and A_1g_ modes of 2H-phase MoS_2_ [37], and the difference between frequencies (Δ) is ≈ 23 cm^−1^, which suggests the thickness is about 4~5 layers [38]. Furthermore, in order to characterize the roughness and to determine the precise thickness of the grown MoS_2_, the surface topography of the AFM image was measured and displays good flatness, presented in Figure 1e, and the height profile along the red line at the edge of MoS_2_ is shown in Figure 1f, the height of which is about *H* = 4 nm, consistent with the Raman result. Finally, TFT devices were fabricated based on this highest-quality patterned MoS_2_ array by laser direct writing lithography. The schematic and physical photos of array devices, and the optical image of the unit device, are shown in Figure 1a,g, respectively.

### 3.2. The Biomodification of the Sensing Part

**Figure 2:**
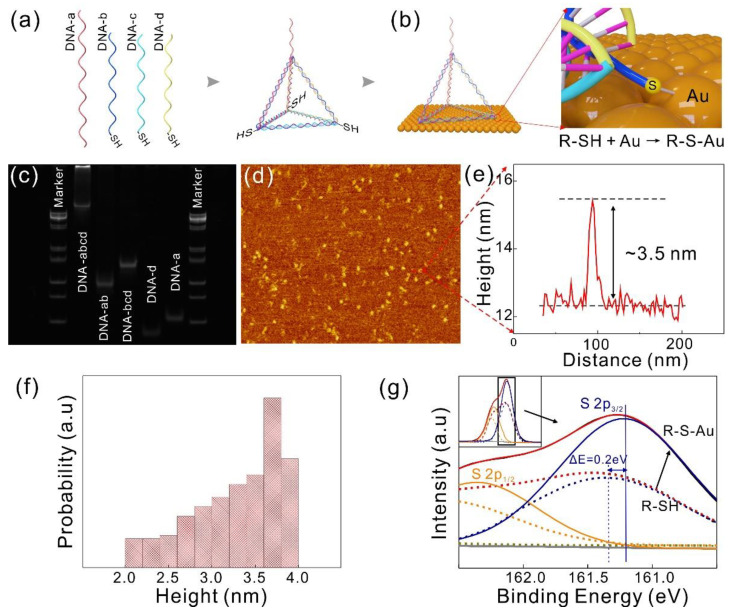
(**a**) Schematic illustration of the synthesis process of DNA tetrahedron probes (DNA-TPs). (**b**) The biomodification schematic of the sensing pad. (**c**) Gel electrophoresis image of the marker and different synthetic strands of DNA sequences. (**d**) Surface topography of DNA-TPs examined by AFM, and (**e**) the height profile along the red line. The height of the DNA-TPs is 3.5 nm. (**f**) Histogram of the probability of the height of DNA-TPs. (**g**) The enlarged X-ray photoelectron spectra of S 2p before (dash lines) and after biomodification (solid lines) according to the insert. The S 2p, S 2p_1/2_, and S 2p_3/2_ peaks are presented in red, yellow, and blue, respectively.

Au nanoparticle immobilization of the single-stranded DNA deposited on the Au electrode could separate the sensing part from TFTs, but the single strand of DNA in this modification method is easily adsorbed on the Au electrode [39,40]. The process of biological modification and target DNA hybridization is full of uncertainties, making the detection results of the device vulnerable to interference. Moreover, the target capture rate of single-stranded DNA is much lower than that of DNA-TPs [41]. In order to improve the stability of the sensing part, the DNA-TPs with a rigid three-dimensional structure are used in the sensing part of biomodification [31]. We have designed four complementary DNA strands to form the DNA-TPs. DNA-a contains the complementary sequence of the target probe—DNA-b, c, d have been modified with thiol groups so that the synthesized tetrahedron is fixed on the surface of the Au detection area through gold–sulfur (Au-S) bonds. A schematic illustration of the structure of the DNA tetrahedron synthesis is shown in Figure 2a, and the specific process can be seen in the Experiment Section, and the detail of TPs are given in Supplementary Materials. In order to verify the quality of DNA-TP formation, polyacrylamide gel electrophoresis (PAGE) and AFM were employed to characterize the synthesized probes. The molecular weights of synthesized DNA from different sequences are shown in Figure 2c. The outermost left and right sides are marked lanes, and the remaining lanes from left to right are DNA-TPs, double-stranded DNA-ab, triple-stranded DNA-bcd, and single-stranded DNA d and a, respectively. DNA-a has more bases than DNA-b (equal to DNA-c and d), which causes the different electrophoresis rate of DNA-a and b, c, d. DNA-TP with four DNA sequences, which has the largest number of bases, shifts slower than others. The AFM image is displayed in Figure 2d, and the surface topography close to the triangle reveals a good tetrahedron structure of DNA-TPs. Moreover, the height profile of the DNA-TPs along the red line is about 3.5 nm (shown in Figure 2e). The height of DNA-TPs ranges from 2 nm to 4 nm, with 3.5~4 nm accounting for the largest proportion; moreover, the average of DNA TPs with standard deviation is about 3.3 ± 0.5 nm (plotted in Figure 2f), which is consistent with the theoretical calculation in the Supplementary Materials. The next crucial step in biomodification is the formation of a self-assembled monolayer (SAM) by the combination of sensing Au pad and DNA-TPs. Among them, the cartoon schematic diagram of the linkage of thiol group and Au is shown in Figure 2b, and the X-ray photoelectron spectra of S 2p is presented in Figure 2g. The S 2p doublet was fitted with two coupled peaks of S 2p_1/2_ and S 2p_3/2_, using Gaussian–Lorentzian peak profiles after the Shirley background subtraction. The energy change (ΔE) from thiol group to Au-S bond of S 2p_3/2_ is ≈ 0.2 eV, which confirmed the successful formation of Au-S bonds [42].

### 3.3. Principle of the Separated Gate-Free Sensor

**Figure 3:**
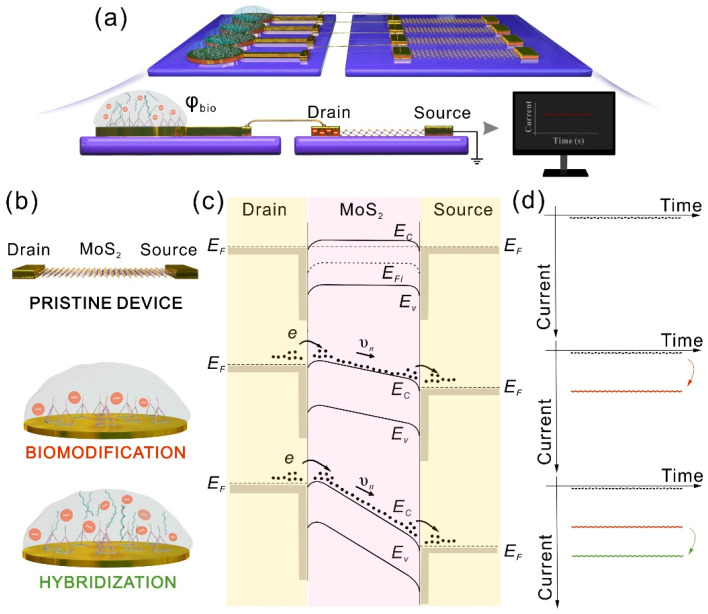
(**a**) Schematic diagram of the bio-separated gate-free sensor array. (**b**) Cartoon illustration of pristine thin film transistor (TFT) device and the changes in the amount of charge after biomodification and hybridization. (**c**) Electron distribution and energy band diagrams between the source/drain electrodes and the MoS_2_. (**d**) Analysis of current changes based on the mechanism in (**c**). *E_F_* is the Fermi level of electrode, *E_Fi_* is the Quasi-Fermi of MoS_2_, and *E_C_* and *E_V_* are the energies of electrons at the conduction band minimum and valence band maximum, respectively. $v_{n}$ represents the average drift velocity of electrons.

After finishing the biomodification, the two separated parts were connected by a conductive Au wire to form the bio-separated and gate-free sensor arrays. The separation of liquid and TFTs makes the detection system have good biocompatibility and satisfies the repeatability of the TFT arrays. The sketch diagram is illustrated in Figure 3a. When the MoS_2_ in the pristine TFTs is in ohmic contact with the source/drain (S/D) electrodes, the electrons in the MoS_2_ could flow extremely to the S/D electrodes. Meanwhile, the electrons in the S/D electrodes could also easily cross the tiny barrier to enter into MoS_2_ [43]. Therefore, the TFTs have a very low dark current in the *I-t* measurement mode, owing to the few-layer semiconductor property of MoS_2_. The related schematic diagrams are shown in Figure 3b–d. Figure 3b visually shows the number of charge changes caused by the biomodification and hybridization by DNA upon the sensing pad. The aqueous solution containing DNA molecules is full of negative charge, since the DNA sequence has a negatively charged phosphate group. The DNA sequences led to the potential change of drain electrode (*φ_d_*, *φ_d_* = *φ_bio_*), hence the *V_ds_* = Δ*φ_bio_*. Figure 3c illustrates the electron distribution and energy band diagram between the drain electrode and MoS_2_. After finishing the biomodification upon the sensing pad, the potential difference between the source and drain electrodes (Δ*φ_ds_*) changes from 0 to Δ*φ_TPs_*, which causes the MoS_2_ energy band to bend. The average drift velocity ($v_{n}$) is directly proportional to the low bio-potential; we might find that

(1) $$v_{n}=-\mu_{n}\Delta \varphi_{ds}/L,$$

where $\mu_{n}$ is the electron mobility. Ignoring the influence of minority carriers (holes), the formula for current could be expressed as

(2) $$I_{ds}=A\cdot \left(-en\right)v_{n}=\frac{WH}{L}\cdot e\mu_{n}n\Delta \varphi_{ds},$$

where A is the channel cross-sectional area, e is the magnitude of electronic charge, and n is the carrier concentration of the MoS_2_. Hence, different concentrations of BRCA1 hybridized with DNA-TPs further increase the Δ*φ_ds_*, which accelerates $v_{n}$ and leads to the increase of the device current. The change of the sensor current corresponding to the energy band structure in Figure 3c is given in Figure 3d.

### 3.4. Detection Performance of the Sensor

**Figure 4:**
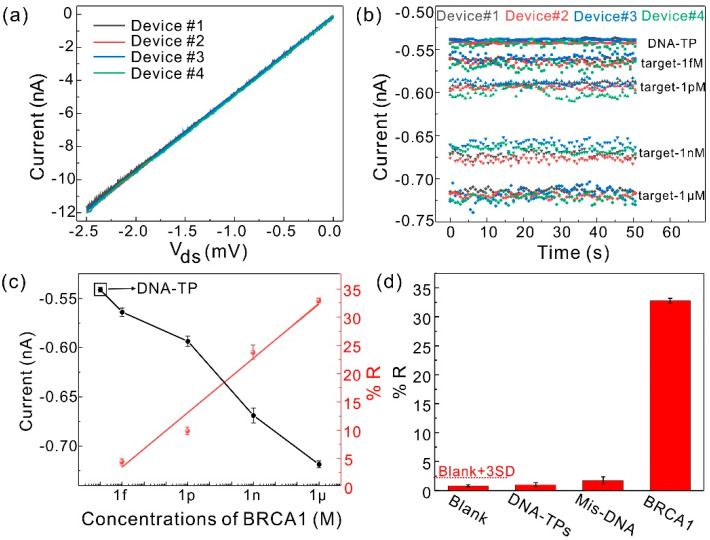
(**a**) Current vs. voltage curves of MoS_2_ TFTs (four devices). Devices #1, #2, #3, and #4 are represented by black, red, blue, and green lines, respectively. (**b**) The stabilized current level over a critical time at different concentrations. Square, circle, regular triangle, inverted triangle, and rhombus represent DNA-TPs, 1 fM, 1 pM, 1 nM, and 1 μM of the target (BRCA1), respectively. (**c**) The currents versus the target concentrations of BRCA1 (the left axis): 0 (DNA-TPs), 1 fM, 1 pM, 1 nM and 1 μM, and linear fitted curve *%R* of the BRCA1 from 1 fM to 1 μM (the right axis). Error bars were calculated from the standard deviation of n = 4. (**d**) The selectivity test of the fabricated sensor array. The concentration of DNA-TPs, mismatch DNA sequence or target DNA (BRCA1) is 1 μM. The dashed line stands for the noise level of the background (blank + 3SD) [41].

The fabricated bio-separated and gate-free sensor arrays for DNA (BRCA1) detection were measured by *I-t*. Before biotarget detection, the electrical performance of the MoS_2_ TFTs was characterized. Figure 4a presents that the current vs voltage (*I_ds_-V_ds_*) curves of MoS_2_ TFT are linear, indicating the excellent ohmic contact of source and drain electrodes with MoS_2_. Moreover, a set of MoS_2_ TFTs array (including four TFT devices) exhibits the almost overlapping *I-V* curves, suggesting superior consistency of the unit device of the detector array. 20 μL DNA-TPs is evenly distributed among the four sensing pads. The current caused by DNA-TPs after completing biomodification is calibrated as the baseline (*I_TPs_*). Then, BRCA1 with different concentrations ranging from 1 fM to 1 μM were successively dropped on the sensing pad of the modified DNA-TPs for hybridization. Figure 4b shows the measured *I-t* curves at different concentrations of BRCA1. The relationship between the response current and different concentrations of target BRCA1 with error bars is shown in Figure 4c of the left axis. Consistent with the previous discussion, the response current increased further with increasing the concentration of BRCA1. In addition, the corresponding plots of response variation (*%R*)

(3) $$\%R=\left|\frac{I_{BRCA1}-I_{TPs}}{I_{TPs}}\right|\times 100,$$

with different concentrations of target BRCA1 are shown in Figure 4c on the right axis; the linear fitted curve of the *%R* shows a good linearity of R^2^ = 0.98. The selectivity of the fabricated sensor was evaluated through comparative experiments of blank, DNA-TPs and a mismatched DNA sequence. The results show that these controlled groups have no obvious *%R* of the BRCA1 in Figure 4d, suggesting the excellent specificity of our proposed sensor.

## 4. Conclusions

We first proposed a novel bio-separated and gate-free structured MoS_2_ TFT biosensor array. The MoS_2_ array was fabricated by a simple-patterned chemical vapor deposition growth method, and the bio-separated sensing part was assembled by a one-step biomodification method based on the DNA tetrahedron probes. The new device structure greatly simplifies the fabrication process. Furthermore, it solves the device biocompatibility (solution erosion) and realizes device reusability. Most importantly, the proposed sensor demonstrated ultrasensitive DNA (BRCA1) detection over a broad range of 1 fM to 1 μM with a good linear response of R^2^ = 0.98. Additionally, the sensor also exhibited excellent specificity against blank and mismatched DNA sequences. However, a good discrimination in a narrow range is difficult to achieve, since the current response caused by the lower concentration change of the target (from 1 fM to 10 fM) is too tiny to distinguish. The shortcoming of the proposed sensor may be solved by replacing MoS_2_ with high-mobility or semi-metallic materials (e.g., multilayer PtSe_2_) [44]. Nevertheless, the bio-separated and gate-free structure sensor arrays with simple fabrication and biomodification manifest a practicable development of high-performance, low-cost, biocompatible, and reusable biosensor systems.

## Figures and Tables

**Table 1:** Performance comparison to other state-of-the-art detection methods for DNA detection.

Detection Technique	Probe	Sensing Range	Detection Limit	REF
FET–biosensor	PNA	1 fM~100 pM	1 fM	[25]
Electrochemical	PNA	10 nM~1 μM	10 nM	[26]
Electrochemical	DNA-TPs	10 fM~10 nM	10 fM	[27]
FET–biosensor	ssDNA	10 pM~10 nM	10 pM	[28]
Electrochemical	ssDNA	10 fg/μL~100 pg/μL	10 fg/μL	[29]
FET–biosensor	ssDNA	0.1 fM~10 fM	0.1 fM	[30]
Electrochemical	DNA-TPs	1 fM~1 nM	0.1 fM	[31]
TFT–biosensor	DNA-TPs	1 fM~1 μM	1 fM	This work

## Data Availability

Data is contained within the article or Supplementary Materials.

## Supplementary Materials

The following are available online at https://www.mdpi.com/2079-4991/11/2/545/s1, Table S1: DNA sequences of tetrahedron probe, Figure S1: Schematic diagram of base complementation of DNA tetrahedron probe synthesis.
