# Supplementary material for: Bio-Separated and Gate-Free 2D MoS2 Biosensor Array for Ultrasensitive Detection of BRCA1
Source: Nanomaterials (Basel). 2021 Feb 21;11(2):545. doi: 10.3390/nano11020545 (PMC7924822; doi:10.3390/nano11020545)
Supplement: Supplementary file 1 [file nanomaterials-11-00545-s001.pdf]

Article

Bio-Separated and Gate-Free 2D MoS_2 Biosensor Array for Ultrasensitive Detection of BRCA1

Yi Zhang [1,2], Wei Jiang [1,2], Dezhi Feng [3], Chenguang Wang [3], Yi Xu [3], Yufeng Shan [4], Jianlu Wang [1], Ziwei Yin [1,2], Huiyong Deng [1,*], Xianqiang Mi [4,5,6,*] and Ning Dai [1, 4,*]

[1] State Key Laboratory of Infrared Physics, Shanghai Institute of Technical Physics, Chinese Academy of Sciences, Shanghai 200083, China; zy_scube@163.com (Y.Z.); 13681827797@163.com (W.J.); jlwang@mail.sitp.ac.cn (J.W.); yinziwei@mail.sitp.ac.cn (Z.Y.)

[2] School of Electronic Electrical and Communication Engineering, University of Chinese Academy of Sciences, Beijing 100049, China;

[3] Shanghai Advanced Research Institute, Chinese Academy of Sciences, Shanghai 201210, China; fengdzh2020@163.com (D.F.); wangcg@sari.ac.cn (C.W.); xuyi@sari.ac.cn (Y.X.)

[4] Hangzhou Institute for Advanced Study, University of Chinese Academy of Sciences, Hangzhou 310024, China; shanyufeng@mail.sitp.ac.cn

[5] CAS Center for Excellence in Superconducting Electronics (CENSE), Shanghai 200050, China;

[6] Key Laboratory of Functional Materials for Informatics, Shanghai Institute of Microsystem and Information Technology, Chinese Academy of Sciences, Shanghai 200050, China;

* Correspondence: hydeng@mail.sitp.ac.cn; mixq@mail.sim.ac.cn; ndai@mail.sitp.ac.cn.

Table S1. DNA sequences of tetrahedron probe.

**DNA-tetra**	**Sequences (from 5′ to 3′)**
DNA-a	ACATTCCTAAGTCTGAAACATTACAGCTTGCTACACGAGAAGAGCCGC CATAGTA TTTTT GATTTTCTTCCTTTTGTTC
DNA-b	HS-TATCACCAGGCAGTTGACAGTGTAGCAAGCTGTAATAGATGCGAGGGT CCAATAC
DNA-c	HS-TCAACTGCCTGGTGATAAAACGACACTACGTGGGAATCTACTATGGCG GCTCTTC
DNA-d	HS-TTCAGACTTAGGAATGTGCTTCCCACGTAGTGTCGTTTGTATTGGACC CTCGCAT

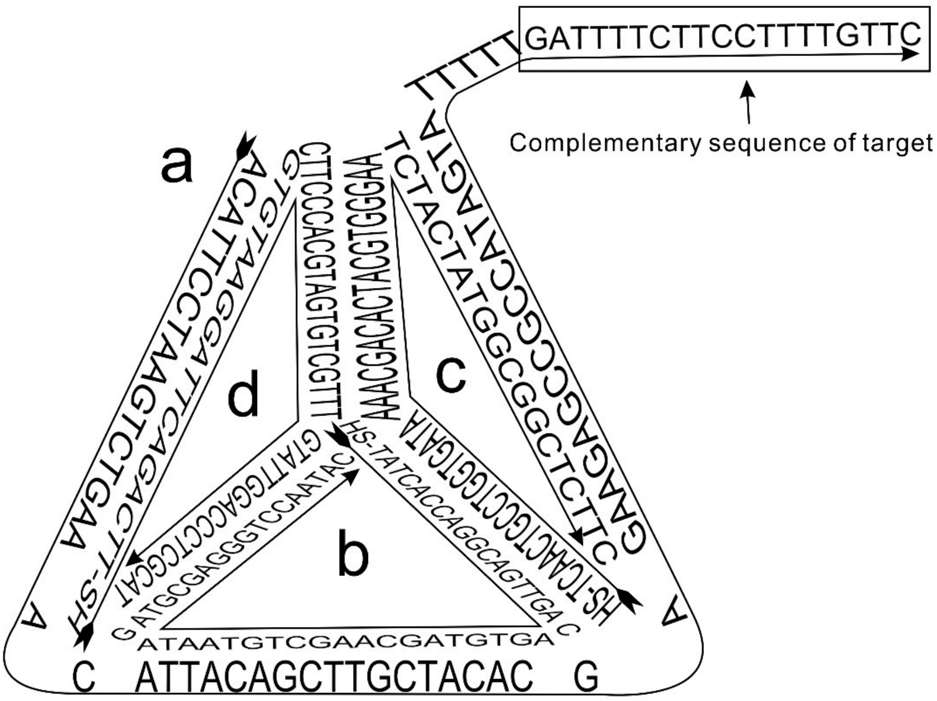

Figure S1. Schematic diagram of base complementation of DNA tetrahedron probe synthesis.

Figure S1 shows the structure diagram of DNA tetrahedral probe synthesis, where the bottom surface of the tetrahedron is composed of an equilateral triangle with a side length of 17 bases (One base length is a, a = 0.34 nm). The height of the tetrahedron is estimated as

$$h = 17a \times \frac{\sqrt{6}}{3}, \quad (1)$$

h ≈ 4.7 nm. And the height of the tetrahedron guaranteed by the experimental data should be less than or equal to 4.7 nm.
